# Right Atrial and Right Ventricular Function Assessed by Speckle Tracking in Patients with Inferior Myocardial Infarction

**DOI:** 10.31083/j.rcm2304123

**Published:** 2022-04-01

**Authors:** Nilda Espinola-Zavaleta, Pablo José Gonzalez-Velasquez, Rodrigo Gopar-Nieto, Gyselle Camacho-Camacho, Enrique Solorzano-Pinot, Valente Fernández-Badillo, Joaquin Berarducci, Javier Ivan Armenta-Moreno, Erick Alexanderson-Rosas

**Affiliations:** ^1^Department of Nuclear Cardiology, National Institute of Cardiology Ignacio Chavez, 14080 Mexico City, Mexico; ^2^Department of Echocardiography, ABC Medical Center I. A. P., 01120 Mexico City, Mexico; ^3^Teaching Department, National Institute of Cardiology Ignacio Chavez, 14080 Mexico City, Mexico; ^4^Department of Physiology, National Autonomous University of Mexico, 04510 Mexico City, Mexico

**Keywords:** right atrial strain, right ventricular strain, inferior myocardial infarction, speckle tracking

## Abstract

**Background:**

In patients with inferior myocardial infarction (MI), involvement of the right chambers has a prognostic impact. The objective of this study was to evaluate the influence of left ventricular (LV) inferior wall MI in the right atrial (RA), and right ventricular (RV) longitudinal strain (LS) by 2D speckle tracking echocardiography (STE).

**Methods:**

60 consecutive patients who underwent myocardial perfusion (MP) gated SPECT for chest pain were included. We studied 30 patients with LV inferior MI and 30 control subjects with normal MP. RV ejection fraction was measured by 3D transthoracic echocardiography, RV-free wall LS and RA reservoir, contraction, and conduit phases strain were analyzed by 2D speckle tracking echocardiography (STE).

**Results:**

The median age in the LV inferior MI was 65 (54–70) years, 27% had a transmural myocardial infarction and 47% had residual myocardial ischemia, most of them, mild (36.7%). RV-free wall LS (–26.1 vs –30.3, *p *< 0.01), RA LS-reservoir phase (31.5 vs 56.2, *p *< 0.01), and RA-conduit phase LS (12.5 vs 35, *p* = 0.01) were significantly lower in the LV inferior MI patients compared to control subjects. In a logistic regression model, the MI of the LV reduced the 3D ejection fraction of both ventricles, mitral regurgitation, and pulmonary hypertension were associated with a decrease in RV LS and RA LS.

**Conclusions:**

This study shows that RV free wall LS, RA peak strain (reservoir phase), and RA conduit phase strain were significantly lower in patients with LV inferior MI vs control individuals. Subclinical extension to the RV in the inferior MI of the LV and its role in the longitudinal strain of RA could be determined using speckle tracking echocardiography.

## 1. Introduction

The prevalence of right ventricular (RV) involvement in acute myocardial infarction (MI) of the left ventricle (LV) ranges from 50 to 80% in postmortem studies [1]. Diagnosis is based on physical examination, electrocardiogram (ECG) with ST-segment elevation in the right precordial leads, and RV dilatation with functional impairment on echocardiogram [2]. Due to the diagnostic limitations of the ECG and conventional 2D echocardiography, smaller RV infarcts and RV involvement are often not detected in the clinical setting [3]. RV involvement in the LV inferior MI is a strong predictor of major complications and in-hospital mortality, as well as long-term morbidity [4].

The right atrium (RA) modulates cardiac output. Rigid ventricles are highly dependent on the atrial contribution for adequate diastolic filling and maintenance of cardiac output. In RV MI, the dilated and rigid ventricle increases the intracavitary pressure against which the RA must empty, therefore it increases its contractility and diastolic filling as a compensatory mechanism [5]. RA ischemia is a strong prognostic marker in the follow-up of patients with RV MI that signifies a greater extension of RV ischemic injury and, consequently the development of a subgroup with significantly high-risk RV dysfunction [6].

Two-dimensional speckle tracking echocardiography (2D-STE) is a useful technique in monitoring, independent of the angle of myocardial deformation that allows a quantitative evaluation of global or regional myocardial function. This method allows the exploration of the RV-free wall deformation and the RA LS during the phases of the cardiac cycle [7].

The strain rate performance improved the diagnostic accuracy for detecting MI. A study showed that RV-free wall strain ≤–14% predicts early mortality in patients with inferior MI (sensitivity–88.9%, specificity–62.5%, positive predictive value–22.9%, negative predictive value–97.8%) [8]. In addition, RV-free wall strain may provide useful information to predict the prognosis of patients with inferior MI. The prognostic value of RA function in inferior MI remains unknown [9, 10]. It is necessary to delve into the role played by right atrial and right ventricular strain and its relationship with inferior MI.

The objective of this study was to assess the LS of the RV-free wall, and the RA LS using 2D-STE of patients with LV inferior MI. It was hypothesized that subclinical extension to RV in LV inferior MI and the role of RA LS could be evaluated using 2D-STE.

## 2. Methods 

Between January and December 2018, we prospectively included 60 consecutive patients who had myocardial perfusion (MP) Gated-Single Photon Emission Computed Tomography (SPECT) due to chest pain and suspicion of coronary artery disease (CAD). We found 30 patients with LV inferior MI and 30 control subjects, who had normal MP Gated-SPECT. The echocardiographic study was performed in all patients using an ACUSON SC2000 System (Siemens, Germany) with a 4V1c transducer (for 2D measurements) and a 4Z1c (for 3D measurements). All echocardiographic measurements were assessed according to the guidelines of the American Society of Echocardiography [11].

### 2.1 Myocardial Perfusion SPECT (Rest/Stress)

A Symbia Siemens, cardiocentric, smartzoom gamma chamber was used (photopeak 20% in 120 keV, matrix 128 × 128, 16 frames). Images were acquired using the following protocol: For rest and rest gated images, 3 mCi 12 seconds/image, and for stress and stress gated images: 9 mCi, 9 seconds/image. Pharmacologic (with dipyridamole) and treadmill stress were performed according to the patient’s physical and clinical characteristics.

### 2.2 Two-Dimensional Echocardiography

Comprehensive two-dimensional (2D) and color Doppler evaluations were performed. The transmitral E/A ratio was determined in the apical four-chamber plane using pulsed-wave Doppler. Peak mitral annular velocity (e’) was measured with tissue Doppler by placing the volume sample in the basal portion of the interventricular septum in the apical four-chamber view. The E/e’ ratio reflects the LV filling pressure. Diagnosis of diastolic dysfunction was made according to the guidelines of the American Society of Echocardiography [12].

RV dimension was measured in the apical four-chamber view at the level of the RV basal cavity at end-diastole; dilatation was considered with a RV basal diameter >42 mm. RV end-diastolic and end-systolic areas, and the fractional area change (FAC) were also measured in the apical four-chamber view. Tricuspid annular plane systolic excursion (TAPSE) was acquired in the apical four-chamber plane with M-mode as a measure of RV longitudinal systolic function.

The RV Tei index was determined as the sum of the isovolumetric contraction time and isovolumetric relaxation time divided by the ejection time, by pulsed-wave Doppler tissue imaging (DTI) in an apical four-chamber view. The S wave was defined as peak systolic velocity of the tricuspid annulus by DTI (cm/sec), in a four-chamber apical view. The criteria for RV systolic dysfunction were: FAC <35%, TAPSE <17 mm, RVMPI >0.54, and S’ <9.5 cm/sec, according to the American Society of Echocardiography guidelines and standards for cardiac chamber quantification by echocardiography in adults published in 2015 [11].

The systolic pulmonary artery pressure (SPAP) was evaluated as the sum of the maximal pressure difference between the right cavities, using color and continuous-wave Doppler, and the mean right atrial pressure was calculated measuring the diameter of the inferior vena cava and its respiratory variation. Estimated SPAP was considered abnormal when the peak tricuspid regurgitation velocity >2.9 m/s, equivalent to >35 mmHg [13].

### 2.3 Three-Dimensional Echocardiography

**Fig. 1.**
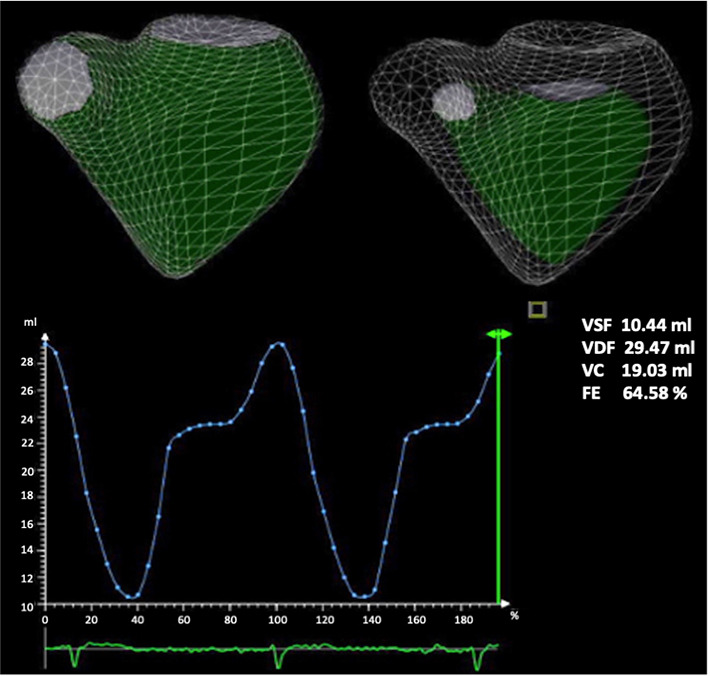
**Right ventricular 3D ejection fraction**. Normal right ventricular EF (64%) in a control subject.

Three-dimensional (3D) left and right ventricular volumes and ejection fraction (EF) were evaluated in the apical four-chamber view focused on the right ventricle for RV analysis (Fig. 1).

Full volume acquisition was performed by ECG activation in three consecutive cardiac cycles during a single apnea. The 3D digital data set was analyzed with commercial software. LVEF was calculated from end-diastolic and end-systolic volumes, LV systolic function was considered abnormal when LVEF <52% for men and <54% for women. An abnormal threshold <45% was considered in RV 3D EF [11].

### 2.4 Two-Dimensional Speckle-Tracking Echocardiography (2D STE)

**Fig. 2.**
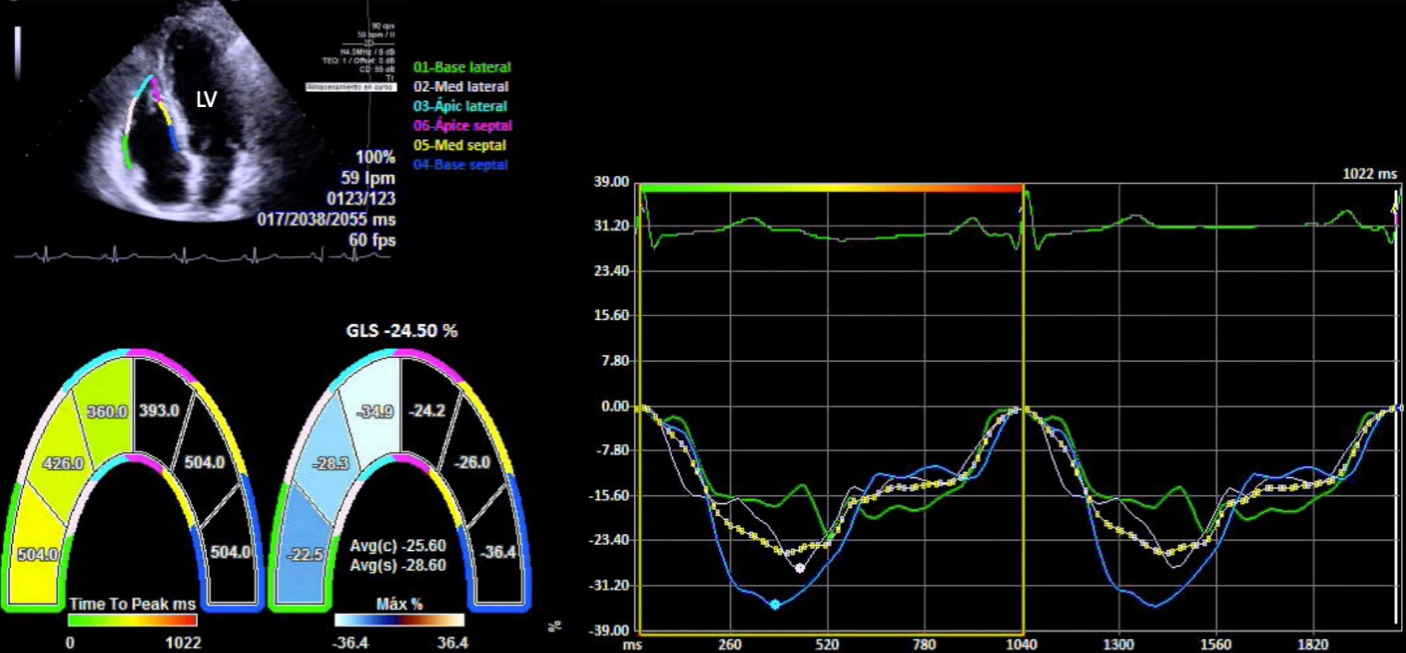
**Free wall longitudinal strain of the right ventricle**. Normal RV-free wall LS (–28.6%). Abbreviations: LV, left ventricle.

The global myocardial strain of the LV, the RV-free wall LS, and the RA LS were measured using the 2D STE analysis. The LV LS was performed in the apical views of four, two, and three chambers and the abnormal value was <–20%. The RV-free wall LS was determined in the apical four-chamber view, focused on the right ventricle, in three consecutive cardiac cycles (>61 frames per second). The region of interest (ROI) was traced with the point-and-click modality in the endocardium at the end of the diastole of the right ventricle. ROI was defined in detail by visual analysis during movie playback, to properly track all segments. After computational analysis, LS was obtained from the RV-free wall (Fig. 2). An abnormal threshold was <–24% [14].

**Fig. 3.**
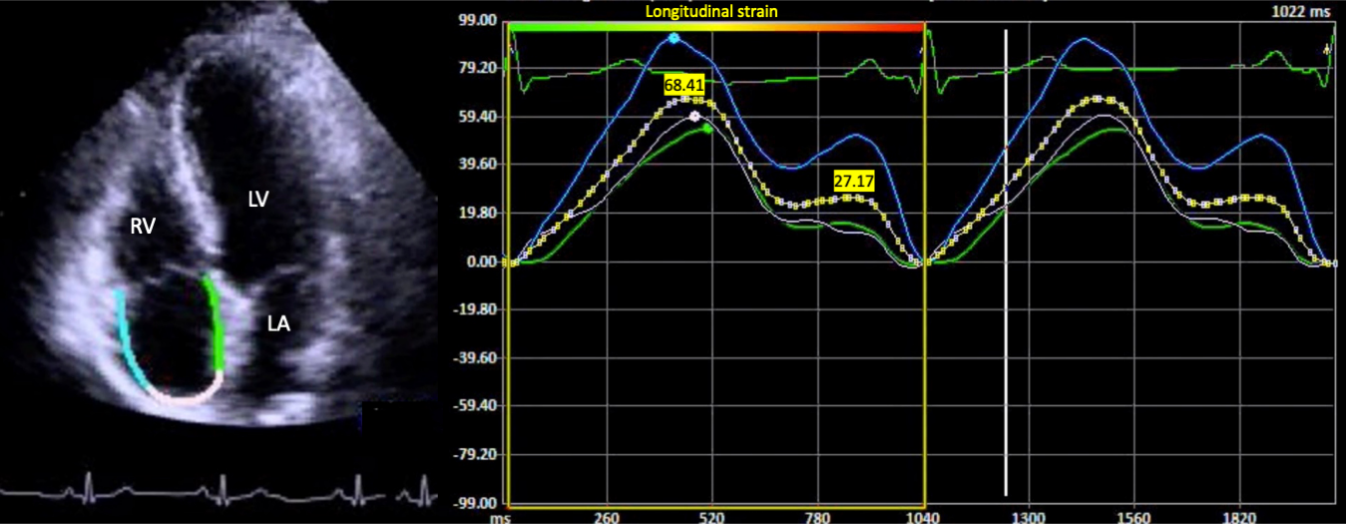
**Right atrial global longitudinal strain**. Normal RA LS with its three phases in a control subject. Abbreviations: LV, left ventricle; LA, left atrium; RV, right ventricle.

RA LS reservoir (peak strain), contraction and conduit phases were assessed in the apical four-chamber view, the QRS-wave was considered as a reference for calculation. The endocardial border was traced manually, delineating a region of interest (ROI), then, from the quality analysis of the segmental tracking and the eventual manual adjustment of the ROI, the software generated the RA LS curves (Fig. 3).

At present, there is no consensus about the reference value for RA peak strain, in 2011, Padeletti *et al*. [15] proposed a mean of 49 ± 13% as a reference value in healthy individuals, therefore, an abnormality threshold of <36% from RA peak strain was used.

Follow-up was made by telephone and patient visits and the primary endpoint was all-cause death.

### 2.5 Statistical Analysis

Data were analyzed with STATA/IC v13 (Stata Corp, College Station, TX, USA). The study was double-blinded, both for echocardiographic analysis and to the SPECT results.

For the descriptive analysis, binary variables were described as frequencies and proportions, and they were analyzed with Pearson’s independence text ($X^{2}$) or Fisher’s exact test, according to the number of individuals per case in the 2 by 2 table. Quantitative variables were analyzed first with Shapiro-Wilk’s normality test, and according to this, they were described as parametric (mean, standard deviation, minimum-maximum) or non-parametric (median, interquartile range, minimum-maximum). Bivariate analysis for parametric variables was made with Student’s *t*-test, and for non-parametric variables, the Mann-Whitney’s test was used. A logistic regression model for determining the risk factors associated with RV and atrial dysfunction was performed. For survival analysis, we used bivariate comparisons of survival proportions with life tables and Kaplan-Meier plots. A *p*-value < 0.05 was considered statistically significant.

## 3. Results

The median age in LV inferior MI group was 65 (54–70) years, 83% were male, 60% of cases had systemic hypertension, and 43% diabetes. 27% had a transmural myocardial infarction and 47% had residual myocardial ischemia, most of them, mild (36.7%). Table 1 demonstrates the clinical findings of the studied groups.

**Table 1.** **Baseline characteristics**.

	LV inferior wall MI patients (n = 30)	Control subjects (n = 30)	*p* value
Age in years (median, IQR)	65 (54–70)	54 (29–58)	0.01
Males (n, %)	25 (83.3)	14 (46.7)	0.03
Females (n, %)	5 (16.7)	16 (53.3)	
Risk Factors			
Diabetes mellitus (n, %)	13 (43.4)	2 (6.6)	<0.01
Hypertension (n, %)	18 (60)	8 (26.7)	<0.01
Dyslipidemia (n, %)	9 (30)	2 (6.7)	0.02
Paroxysmal AF (n,%)	3 (10)	0	0.11
Smoking (n, %)	11 (36.7)	1 (3.3)	<0.01
CKD (n, %)	2 (6.7)	0	0.24
Infarction characteristics			
RV extension (clinical) (n, %)	1 (3.3)	0	0.5
Transitory AV block (n, %)	3 (10)	0	0.11
Mechanical complication (n, %)	2 (6.7)	0	0.24
SPECT characteristics			
Transmural infarction (n, %)	8 (26.7)	0	<0.01
Residual ischemia			
Mild (n, %)	11 (36.7)	0	<0.01
Moderate (n, %)	2 (6.7)	0	0.24
Severe (n, %)	1 (3.3)	0	0.5

LV, left ventricle; MI, myocardial infarction; IQR, interquartile range; AF, atrial fibrillation; CKD, chronic kidney disease; RV, right ventricle; AV, atrioventricular; SPECT, single photon emission computed tomography.

Table 2 describes the echocardiographic measurement of the studied population. In LV inferior MI, a larger LV end-diastolic volume (121 vs 89.5 mL, *p *< 0.01), a lower 3D LV ejection fraction (46% vs 60%, *p *< 0.01), an increased medial E/e’ (9.59 vs 7.7, *p *= 0.01) and a lower RV systolic function measurements (18.6 vs 21.7 mm for TAPSE, *p *< 0.01; 37% vs 46.9% for FAC, *p *< 0.01; 9.9 vs 11.8 cm/s for S’, *p *< 0.01; and 47.5% vs 54% for 3D EF, *p *< 0.01) were found. LV peak global LS (–13.3 vs –22%, *p *< 0.01), RV free wall LS (–26.1 vs –30.3 %, *p *< 0.01), RA LS-reservoir phase (31.5 vs 56.2%, *p *< 0.01) and RA LS conduit phase (12.5 vs 35%, *p *= 0.01) were significantly lower in the LV inferior wall MI patient’s vs control subjects.

**Table 2.** **Echocardiographic parameters**.

	LV inferior wall MI patients (n = 30)	Control subjects (n = 30)	*p* value
3D LV EDV (mL) (median, IQR)	121 (99.7–150)	89.5 (73–99)	<0.01
3D LV EF (%) (median, IQR)	46 (37–53)	60 (58–63)	<0.01
Mitral E/A (median, IQR)	0.82 (0.7–1.2)	1.21 (0.97–1.44)	<0.01
Medial E/e’ (median, IQR)	9.59 (7.9–13)	7.7 (6.9–10.5)	0.01
RV EDD (mm) (mean, SD)	37.6 ± 7.4	34.7 ± 4.39	0.12
TAPSE (mm) (mean, SD)	18.6 ± 5.05	21.7 ± 4.1	<0.01
RV FAC (%) (mean, SD)	37.01 ± 9.06	46.86 ± 7.24	<0.01
RV S’ (mean, SD)	9.92 ± 2.68	11.78 ± 1.85	<0.01
3D RV EF (%) (median, IQR)	47.5 (42–52)	54 (49–57)	<0.01
RIMP (mean, SD)	0.49 ± 0.18	0.46 ± 0.12	0.60
SPAP (mmHg) (median, IQR)	31.5 (27.37)	29.5 (26.33)	0.07
Speckle tracking analysis			
Peak global LV LS (%) (median, IQR)	–13.35 (–17.6, –9.3)	–22 (–23.2, –20.4)	<0.01
Peak free wall RV LS (%) (median, IQR)	–26.1 (–32.1, –17.8)	–30.3 (–36.6, –27.5)	<0.01
RA peak strain (reservoir phase) (%) (median, IQR)	31.5 (25.2–43)	56.2 (47–66.4)	<0.01
RA contraction phase strain (%) (Mean, SD)	20.17 ± 10.39	24.4 ± 10.36	0.07
RA conduit phase strain (%) (Median, IQR)	12.5 (5.9–15.6)	35 (21–47.6)	0.01

LV, left ventricle; MI, myocardial infarction; 3D, three dimensional; EDV, end-diastolic volume; ml, milliliters; IQR, interquartile range; EF, ejection fraction; RV, right ventricle; EDD, end-diastolic diameter; mm, millimeters; SD, standard deviation; TAPSE, tricuspid annular systolic plane excursion; FAC, fractional area change; S’, tissue Doppler imaging of peak systolic tricuspid annulus; RIMP, right ventricle index of myocardial performance; SPAP, systolic pulmonary artery pressure (estimated); LS, longitudinal strain; RA, right atrium.

LV inferior MI [Odds ratio (OR) 4.3, 95% CI 1.05–17.85, *p *= 0.04], reduced 3D LV EF (OR 4.53, 95% CI 1.23–16.58, *p* = 0.02), and mitral regurgitation (OR 5.1, 95% CI 1.3–19.5, *p *= 0.01) were significantly associated with reduced RV free wall LS (<–24%) in the logistic regression model. We also found a significant association between RV dilatation (OR 7.02, 95% CI 1.7–29.4, *p *< 0.01), reduced TAPSE (OR 9, 95% CI 2.01–40.2, *p *< 0.01), and elevated SPAP (OR 16.8, 95% CI 3.7–76.6) with reduced RV free wall LS (Table 3).

**Table 3.** **Clinical and echocardiographic parameters associated with altered RV Free Wall LS**.

	OR	95% CI	*p* value
LV inferior wall MI	4.3	1.05–17.9	0.04
Decreased 3D LV EF	4.5	1.2–16.6	0.02
Mitral regurgitation	5.1	1.35–19.5	0.01
RV dilatation (basal diameter >42 mm)	7.02	1.7–29.4	<0.01
Decreased TAPSE (<17 mm)	9	2.01–40.2	<0.01
Decreased 3D RV EF (<45%)	4.8	1.2–18.5	0.02
Elevated SPAP (>35 mmHg)	16.8	3.7–76.6	<0.01

OR, odds ratio; CI, confidence interval; LV, left ventricle; MI, myocardial infarction; 3D, three dimensional; EF, ejection fraction; RV, right ventricle; TAPSE, tricuspid annular systolic plane excursion; SPAP, systolic pulmonary artery pressure (estimated).

When patients were divided according to RA peak strain (reservoir phase) cut-off value of 36%, LV inferior wall MI was significantly associated with reduced RA peak strain (OR 10.8, 95% CI 2.97–39.2, *p *< 0.01). Smoking (OR 4.3, 95% CI 1.1–16.42, *p* = 0.03), reduced 3D LVEF (OR 7.7, 95% CI 2.3–26.14, *p *< 0.01), mitral regurgitation (OR 9.5, 95% CI 2.8–31.8), abnormal S’ (OR 4.8, 95% CI 1.4–16.7, *p *= 0.01), reduced 3D RV EF (OR 15.6, 95% CI 3.01–80.6, *p *< 0.01) and elevated SPAP (OR 4.3, 95% CI 1.1–16.4, *p *= 0.03) were also associated with reduced RA LS (Table 4).

**Table 4.** **Clinical and echocardiographic parameters associated with altered RA peak strain (reservoir phase)**.

	OR	95% CI	*p* value
LV inferior wall MI	10.8	2.97–39.2	<0.01
Smoking	4.3	0.36–5.21	0.03
Decreased 3D LV EF	7.8	2.3–26.1	<0.01
Mitral regurgitation	9.5	2.8–31.8	<0.01
Abnormal S’ (<9.5 cm/s)	4.76	1.4–16.7	0.01
Decreased 3D RV EF (<45%)	15.6	3.01–80.6	<0.01
Elevated SPAP (>35 mmHg)	4.3	1.1–16.4	0.03

OR, odds ratio; CI, confidence interval; LV, left ventricle; MI, myocardial infarction; 3D, three dimensional; EF, ejection fraction; S’, tissue doppler imaging of peak systolic tricuspid annulus; RV, right ventricle; SPAP, systolic pulmonary artery pressure (estimated).

A 4-year follow-up was performed, with special attention to survival, subsequent surgical procedures (stent or bypass), NYHA functional class, and deaths. We found that in the group of cases, three patients suffered a reinfarction and one of them died. In these patients, the mean values of the RA peak reservoir strain and LV LS in the baseline echocardiogram were reduced to 30% and –11.09%, respectively. In the patient who died after myocardial reinfarction, RV-free wall LS (–13.6%) was decreased. In the control group, one patient had an inferior MI, despite normal biventricular LS values.

## 4. Discussion

Our study found that RV-free wall and RA LS were significantly reduced in patients with LV inferior MI compared with control individuals. Previously published studies have demonstrated that RV LS measured by STE may be a valid method for the evaluation of the RV function in multiple clinical scenarios [16, 17, 18], including right coronary artery disease [18], first inferior wall MI [19], and right ventricular MI [20]. It also has prognostic information that is superior to conventional echocardiographic measures [21, 22].

STE also has been used to perform LV strain rate and its value is being applied with favorable results in the diagnosis and prognosis of clinical outcome, LV remodeling, and cardiotoxicity. In addition, speckle tracking has already proven to be a useful prognostic tool for predicting major cardiovascular events in patients with CAD; however, the use of RV-free wall strain and RA LS to predict major cardiovascular events after an inferior MI is still unknown [23, 24, 25].

The mechanism of RV dysfunction after left ventricular MI is not well established, but it is often presumed that failure of the LV provokes pulmonary hypertension and increment of the RV afterload, which leads to RV remodeling and dysfunction. In 2013, Konoshi K *et al*. [26], found that RV LS depends on LV systolic function in patients with old inferior wall MI; we found a significant association between decreased 3D LVEF and elevated SPAP, with reduction of RV free wall LS. We also found a significant association between RV dilatation and systolic dysfunction (measured by 3D RV EF and TAPSE) with reduced RV free wall LS. The involvement of RV and/or septum by myocardial infarction or ischemia are common in patients with LV MI, and it is a possible mechanism leading to RV systolic dysfunction and dilatation [27].

RA has an important role in RV filling (1) it acts as a reservoir for venous return, (2) as a passive conduit in early diastole, and (3) as reinforcement in end-diastole during atrial contraction [28]. A significant reduction in RA maximum deformation, as a function of RA reservoir phase, was also found in the inferior LV MI group. Furthermore, RA conduit function was lower in this group of patients. These data demonstrate that in patients with left ventricular inferior wall MI, the reservoir and conduit function of the RA was impaired.

Previously Nourian S *et al*. [29], found reduced RA reservoir values (mean value of 26.6%) and conduit phase strain in patients with inferior MI and right ventricular involvement compared with those without right ventricular extension. Our study group was compared with control subjects, the median strain value for the RA reservoir phase and the conduit phase was reduced, but the booster function was preserved (atrial contraction was found to increase in the presence of ventricular systolic dysfunction) [30].

Ventricular function is an important determinant of atrial function. RA reservoir function during early ventricular systolic time is related to RV systolic function, due to longitudinal contraction of the ventricle and downward pull of the base of the ventricle [31]. Our study found a significant association between reduced 3D RVEF and S’ with abnormal RA peak strain (reservoir phase). We also found an association between smoking and reduced RA peak strain, it is known that smoking can induce atrial fibrosis through nicotine [32], and acute consumption can increase afterload due to transitory diastolic dysfunction and increased systolic pulmonary artery pressure [33].

At follow-up, we found that in the patients who developed reinfarction and death, the mean values of the RA peak reservoir strain, LV LS, and RV-free wall LS were reduced at baseline echocardiogram. However, we need a longer follow-up and a larger sample to determine a significant prognostic value.

## 5. Limitations

The data presented here were obtained from a small group of patients, at a single center, referred for clinically indicated myocardial perfusion SPECT. The global strain was measured only in the longitudinal direction, the radial strain may provide more evidence. Strain in LV inferior wall MI and in RV infarction needs extensive validation studies. We use some not well standardized cutoff values in the RA strain parameters.

## 6. Conclusions

RV free wall LS, RA peak strain (reservoir phase), and RA conduit phase strain were significantly lower in patients with LV inferior MI than in control individuals.

The subclinical extension to the RV in LV inferior MI and its role in the longitudinal strain of RA could be determined using speckle tracking echocardiography.
